# Supplementary material for: Comparative multiomics analysis of cell physiological state after culture in a basket bioreactor
Source: Sci Rep. 2022 Nov 23;12:20161. doi: 10.1038/s41598-022-24687-4 (PMC9686226; doi:10.1038/s41598-022-24687-4)

3666038.1:33457205:33457265:+@NW_023666038.1:33457205:33457279:+@NW_023666038.1:33459581:3345

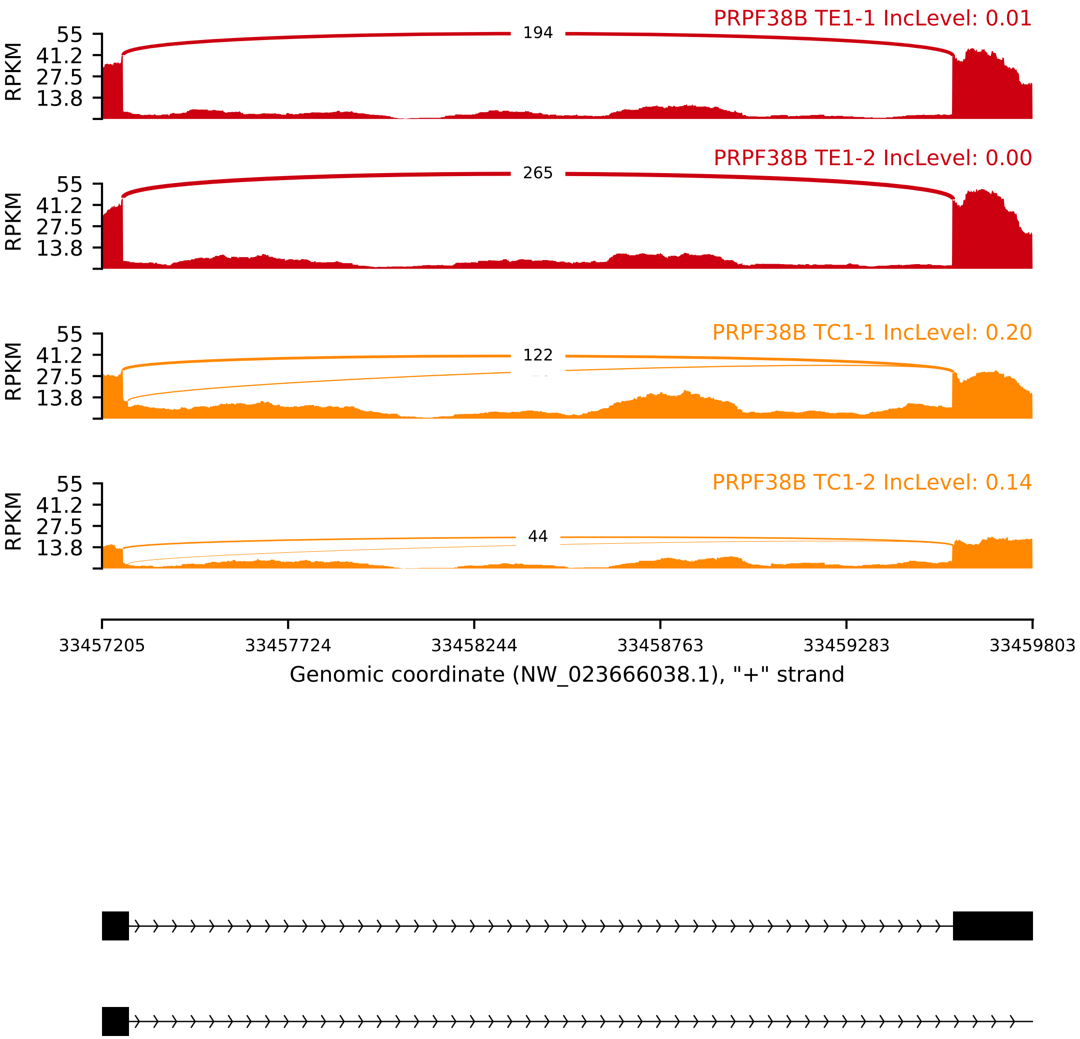

Supplement: Supplementary file 1 — Supplementary Information 1. [file 41598_2022_24687_MOESM1_ESM.zip › raw data/Transcriptomic raw data/7.AS/2.dasplot/TE1vsTC1/A5SS/15_PRPF38B_NW_023666038.1_33457205_33457265_+@NW_023666038.1_33457205_33457279_+@NW_023666038.1_33459581_33459804_+.pdf]
